# Hospital Abuse and Medical Discontent

**Published:** 1906-11-24

**Authors:** 


### The Hospital

A Journal of

The Medical Sciences and Hospital Administration.

Vol. XLI.—No. 1,053. SATURDAY, NOVEMBER 24, 1906.

### Hospital Abuse and Medical Discontent.

The article we published on this subject on the 10th instant is attracting a good deal of attention. The *British Medical Journal* expresses a wish to hear the views of any chairman of the board of a large hospital on the subject of the extent to which hospital committees are in the hands of their visiting medical staffs. The practice in regard to this matter is not uniform so far as the hospitals are concerned. At the London Hospital, for example, no members of the medical staff are eligible to serve on the managing committee, but at most other hospitals the visiting staffs are represented in the management. Our point is, there is cause to fear, for various reasons, that the members of the visiting staffs of the voluntary hospitals have never yet energetically exercised, all their influence continuously to cut down and restrict the amount of free medical relief given by the hospitals, which shows a tendency to largely increase year by year. It must be apparent to everyone, that the abolition of tickets has placed the visiting medical staffs and their juniors in a position, to control the admission or rejection of patients, to an extent, never possible for them, when the ticket system was strictly enforced. As we stated, in our last issue, there are many interests involved in the solution of the problem presented by the excessive free medical relief given to the public by the hospitals at the present time. Our view is, that, if the members of all the visiting staffs can be moved, to exert themselves to the utmost to reduce the number of patients, to an adequate extent, such a reduction can be brought about, with great benefit to the patients, with justice to the medical profession, with increasing efficiency in the quality of the work done by the hospitals for the really deserving poor, and with no small benefit to the reputation of all concerned.

Another point raised by the *British Medical Journal* is, the effect which the existence of large subscriptions from the working classes, with a consequent representation of those classes on the boards of hospitals, has on any attempt to curtail relief. It may be pointed out, that, in fact, the contributions from workpeople, in London, including Hospital Saturday, only amount to about 1½ per cent. of the total ordinary income of the general hospitals, and to but 2½ per cent. of the ordinary income of the special hospitals. The total contributions to the whole of the Metropolitan, Provincial, Scotch and Irish hospitals, including Hospital Saturday, are less than 12 per cent. of their total ordinary incomes, and in the case of special hospitals, they are less than 3½ per cent. These figures show, that in London, the contributions of the working classes are so small, as to be relatively unimportant. Although the percentage is higher in the Provinces, Scotland and Ireland, the average working class contribution to all hospitals is considerably under ten per cent. of their total ordinary incomes. Again, experience proves, that the representatives of the working classes who have served on hospital committees, are strongly in favour of the removal of hospital abuses. They would, we are confident, be found amongst the warmest supporters of any reform, in the direction of reducing the present excessive amount of free medical relief in London, especially, and indeed wherever it may be found to exist in other parts of the country. The working classes are fully alive to the importance of thrift, and they realise, as we do, that the present excessive free medical treatment at the hospitals tends to diminish thrift, and to demoralise the character of the people who habitually receive it.

Two other points have been brought out by the discussion raised by our previous article. One is that the public do not grasp, at present, that hospital abuse is not limited to the gift of free medical service to those who can afford to pay for it. It is an equal abuse for the voluntary hospitals to attend thousands of people for whom the poor-law system makes provision; to admit to treatment very many patients who, in fact, have no direct claim whatever upon London hospitals, and to give medicine to, or to admit to treatment, multitudes of cases, which, from their trivial nature, might well be provided for in other ways. Again, it has been said, that the figures we quoted are unreliable. Now, they are taken from the census returns, and from the figures which appear in the reports of 94 voluntary hospitals, situated in the county of London, and of the hospitals located in the outer ring constituting Greater London. Their fullness and importance must be recognised by all who will take the trouble to refer, to the tables and facts contained in Chapter I. of Burdett's "Hospitals and Charities" for the years 1905 and 1906, respectively. These figures, so far as the numbers of patients are concerned, are the same figures as those upon which the authorities of the hospitals have relied when issuing their appeals to the public for further and larger contributions. It follows, that, if these figures are unreliable, the responsibility must rest with the hospital authorities. But we are confident, from our knowledge of the ability and conscientiousness of the chief officials of these institutions, that the returns have, as a whole, been prepared with considerable care, and that they may be relied upon as being as accurate as they can be made. In such circumstances, it cannot be denied that for 30 years, the growth of free medical relief, given by the voluntary hospitals in the metropolis, has been far in excess of what it ought to have been. Such a state of affairs is inconsistent with the best interests of the hospitals, of their supporters, of the members of the medical profession, and of the public generally.

